# Expression of survivin, a novel inhibitor of apoptosis and cell cycle regulatory protein, in pancreatic adenocarcinoma

**DOI:** 10.1038/sj.bjc.6600133

**Published:** 2002-03-18

**Authors:** A I Sarela, C S Verbeke, J Ramsdale, C L Davies, A F Markham, P J Guillou

**Affiliations:** Academic Unit of Surgery, School of Medicine, University of Leeds, Leeds, UK; Academic Unit of Molecular Medicine, School of Medicine, University of Leeds, Leeds, UK; Department of General Surgery, St James's University Hospital, Leeds LS9 7TF, UK; Pathology, St James's University Hospital, Leeds LS9 7TF, UK

**Keywords:** pancreatic neoplasms, inhibitor of apoptosis, P53, BCL-2

## Abstract

Survivin is unique for its expression in human malignancies but not in normal adult cells. It has been implicated in sensitisation to chemotherapy and as a prognostic marker in several common cancers. Immunohistochemistry for Survivin, P53 and BCL-2 expression as well as cell proliferative index (Ki-67) and apoptosis index (TUNEL) was conducted on 52 pancreatic and 12 ampullary adenocarcinomas. Survivin was detected in the cytoplasm of carcinoma cells in 46 (88%) of pancreatic tumours. P53 and BCL-2 were detected in 54% and 12% of pancreatic tumours, respectively. Proliferative index was 26.2±10.5% and apoptosis index was 1.38±0.69%. Prevalence of Survivin expression was significantly higher in P53-positive than in P53-negative cases (*P*=0.05) but was not associated with BCL-2 expression. Incrementally higher weighted scores of Survivin expression were associated with increased proliferative index (*P*=0.001). Furthermore, there was linear correlation between increased proliferative index and higher apoptosis index (*P*<0.001). Surprisingly, higher scores of Survivin expression were associated with increased apoptosis index (*P*=0.007). Survival characteristics were not influenced by Survivin, P53 or BCL-2 expression, apoptosis index or proliferative index. Ampullary carcinoma showed Survivin expression in 83% of cases. However, unlike pancreatic carcinoma, there was no correlation between Survivin and P53 expression or proliferative index. In conclusion, Survivin is expressed in the majority of pancreatic adenocarcinomas and correlates with both cellular proliferation and apoptosis. Molecular manipulation of Survivin expression may enhance chemotherapy and radiation therapy for pancreatic cancer.

*British Journal of Cancer* (2002) **86**, 886–892. DOI: 10.1038/sj/bjc/6600133 www.bjcancer.com

© 2002 Cancer Research UK

## 

Adenocarcinoma of the pancreas continues to be associated with a dismal prognosis despite substantial improvements in the surgical technique of pancreaticoduodenectomy. Indeed, routine consideration for neoadjuvant or adjuvant chemoradiation is currently recommended (Di Magno *et al*, 1999). There have been enormous advances in potential applications of molecular technology to the treatment of pancreatic cancer (Manu *et al*, 2000) and, for example, gene therapy and anti-angiogenesis therapy appear promising modalities (Humphreys *et al*, 1999; Van Riel *et al*, 1999). Detailed understanding and careful manipulation of cellular processes, such as programmed cell death (apoptosis) or cell proliferation, are fundamental to the application of either chemoradiation or molecular technologies to clinical practice (Fisher, 1994). Not surprisingly, therefore, oncogenes and tumour-suppressor genes, such as P53, as well as the underlying genetic aberrations in pancreatic cancer have been the subject of intense research (Schleger *et al*, 2000).

The inihibitor of apoptosis protein (IAP) and BCL-2 families are critically important in the regulation of apoptosis (Jaattela, 1999). Survivin is a recently described IAP that is unique for its expression in a wide range of embryonic and foetal tissues but is undetectable in terminally differentiated normal adult tissues. However, it is prominently re-expressed in several human cancers (Ambrosini *et al*, 1997). In addition to its function as an inhibitor of apoptosis, Survivin is involved in the regulation of cellular proliferation (Li *et al*, 1999) and angiogenesis in cancers (O'Connor *et al*, 2000). The prognostic value of Survivin expression has been reported in several human cancers (Altieri *et al*, 1999); for example, the authors have recently reported that death from recurrent colorectal cancer is predicted by the detection of Survivin expression using either reverse transcriptase – polymerase chain reaction (Sarela *et al*, 2000) or immunohistochemistry (Sarela *et al*, 2001). Furthermore, a potential therapeutic role is evident from studies demonstrating resistance of Survivin-transfected cells to anticancer drug-induced apoptosis (Tamm *et al*, 1998) and sensitisation to chemotherapy by Survivin antisense treatment (Olie *et al*, 2000).

This study aimed to examine the expression and potential prognostic value of Survivin, as well as P53, BCL-2 and markers of the cellular process of apoptosis and proliferation, in pancreatic and ampullary cancers.

## MATERIALS AND METHODS

### Tissue Samples

Patients who had undergone pancreaticoduodenectomy between 1994–2000 were identified and archival tissue blocks of resected specimens were retrieved. Representative sections were stained with H&E in order to confirm the histopathological diagnosis, and further studies were conducted on 52 adenocarcinomas of ductal origin (excluding intra-ductal papillary mucinous neoplasms) that were identified as originating from head, neck or uncinate process of the pancreas and 12 carcinomas of ampulla of Vater. Pre-operative staging had been conducted using contrast enhanced dual-phase helical CT or MR imaging, as described previously (Sheridan *et al*, 1999). Pathological re-staging for the tumour (T) and lymph node (N) categories was conducted according to the 1997 AJCC classification for pancreatic and ampullary cancer. Grade of differentiation (Luttges *et al*, 2000) and status of the resection margin (R1 if malignant cells within 1 mm of any margin) were recorded for each case. Patient survival data were obtained from the database of the regional cancer registry (Northern & Yorkshire Cancer Registry & Information Service, Leeds, UK).

## IMMUNOHISTOCHEMISTRY

Immunohistochemical stains were performed on paraffin-embedded formalin-fixed tissue sections with a StreptABComplex/HRP detection system (Dako) using DAB/hydrogen peroxide as a chromogen. The mono- and polyclonal antibodies (Mabs, Pabs) used included Survivin (Pab; Autogen Bioclear, Wiltshire, UK; dilution 1 : 200), P53 (Mab; Dako, clone DO-7; dilution 1 : 100), BCL-2 (Mab; Dako; dilution 1 : 40), and Ki-67 (Mab; Dako; dilution 1:50). Antigen retrieval by microwave pretreatment (2×5 min in 0.1 M citrate buffer pH=6 at 600 W) was performed for all Abs except for staining with anti-BCL-2 which required pressure cooker pretreatment (1 min in 10^−3^ M sodium citrate buffer pH=6). Incubation with the primary Abs was done at room temperature for 60 min (P53, BCL-2, Ki-67) or overnight (Survivin). As a negative control, sections were processed in the absence of primary Ab. Tissue sections from a colonic carcinoma with known strong expression of Survivin, P53 and BCL-2 (Sarela *et al*, 2001) were used as a positive control.

### TUNEL

Free DNA-ends created by apoptosis were identified by TdT (terminal deoxyribonucleotidyl transferase)-mediated tailing with digoxigenin-labelled nucleotides and subsequent detection via a peroxidase-conjugated anti-digoxigenin antibody, using the ApopTag kit (Intergen Company, Oxford, UK) as recommended by the manufacturer.

### Semi-quantitative assessment and statistical analysis

Proliferative and apoptotic activity was assessed by scoring the percentage of labelled cells in at least 1000 tumour cells, as previously described (Verbeke *et al*, 2000). Semi-quantitative assessment of Survivin expression was based on the mean percentage of positive tumour cells in at least five high-power fields (×400) assigned to one of five categories: (a) 0=<5%; (b) 1=5–25%; (c) 2=25–50%; (d) 3=50–75%; (e) 4=>75%. The intensity of Survivin immunostaining was scored as (a) weak=1+; (b) moderate=2+; (c) intense=3+. The percentage of positive tumour cells and staining intensity were multiplied to produce a weighted score for each case (Lu *et al*, 1998). In tumours displaying heterogeneous staining, the predominant pattern was considered for scoring. Tumours with a weighted score=0 were designated as negative; all others were considered positive. Sections were scored as positive for P53 or BCL-2 when >10% of tumour cells displayed nuclear or cytoplasmic immunostaining, respectively. The software package SPSS 9.0 was used for statistical analysis. Proliferative index and apoptosis index are reported as mean±standard deviation. Association between Survivin expression and various clinical and pathological variables was examined using the χ^2^ test or Fisher's exact test. The Pearson test was used to examine linear correlation between Survivin weighted indices and proliferative or apoptotic indices. Survival characteristics were studied by the method of Kaplan–Meier and the Cox proportional hazards regression model was used to examine the predictive values of individual variables. Patients who had died of non-cancer related causes and those who were alive at last follow-up were treated as censored observations. *P* value less than 0.05 was considered statistically significant.

## RESULTS

### Clinical and pathological characteristics

Patients were of mean age 64 (57–71) years. Details of clinical and various pathological variables for pancreatic cancer are shown in Table 1

**Table 1:**
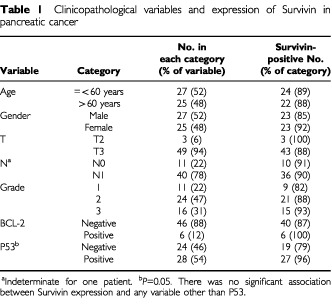
Clinicopathological variables and expression of Survivin in pancreatic cancer

. There was no significant association between the expression of Survivin and gender, discriminators of pathological stage or grade of pancreatic cancer. Ampullary cancers comprised T2 (three), T3 (seven) or T4 (two) tumours that were N0 (three) or N1 (nine) and there was no stage-specific correlation with Survivin expression (data not shown).

### Expression of Survivin

**Figure 1:**
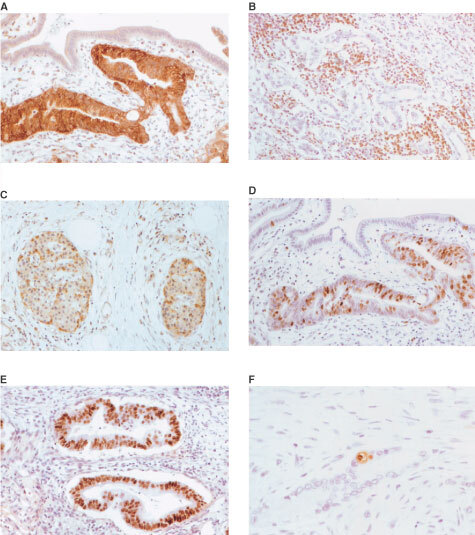
Immunohistochemistry for Survivin, P53, BCL-2, Ki-67 and apoptosis (TUNEL assay) on pancreatic carcinomas. (**A**) Carcinomatous glands show strong cytoplasmic expression of Survivin whereas normal duct epithelium and stromal cells are negative (200×). (**B**) Endocrine cells of Langerhans islets show strong Survivin expression (100×). (**C**) Strong nuclear immunostaining for P53 is seen in neoplastic cells only (200×). (**D**) BCL-2 immunostaining is negative in neoplastic glands. Tumour-infiltrating lymphocytes show strong BCL-2 expression (100×). (**E**) Ki-67 immunostaining reveals high proliferative activity in neoplastic glands whereas only few positive cells are found in normal duct epithelium (200×). (**F**) TUNEL assay detects an apoptotic body flanked by an early apoptotic cell in a neoplastic gland (200×).

**Figure 2:**
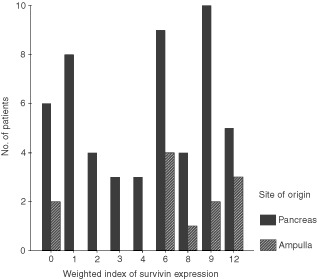
Distribution of weighted scores for Survivin expression in pancreatic and ampullary cancers.

Immunohistochemistry for Survivin revealed granular staining in the cytoplasm of pancreatic carcinoma cells (Figure 1

). A few tumours showed nuclear staining in a minority of cells. Similar features were observed in ampullary cancers. Whilst no expression of Survivin was detected in either the stromal cells of the tumours or in the adjacent non-neoplastic ductal or acinar epithelium, there was strong reproducible staining of endocrine cells within the islets of Langerhans (Figure 1). Weighted scores for Survivin expression varied from 0–12 (Figure 2

). Using a weighted score=1 for stratification, 46 (88%) pancreatic carcinomas and 10 (83%) ampullary carcinomas were Survivin-positive.

### Expression of P53 and BCL2

Some 28 (54%) of pancreatic cancers were P53 positive. Nuclear immunostaining for P53 was limited to carcinoma cells and was not observed in any stromal cells or adjacent non-neoplastic epithelium (Figure 1). There was a significant association between expression of Survivin and that of P53 (Table 1). Six (12%) pancreatic cancers were BCL-2 positive and demonstrated staining in the cytoplasm of carcinoma cells. Infiltrating stromal lymphocytes also stained for BCL-2, irrespective of the BCL-2 status of the cancer cells, and served as positive internal controls (Figure 1). There was no correlation between expression of Survivin and that of BCL-2. Of ampullary cancers, eight were P53-positive and only one was BCL-2 positive; there was no association with Survivin expression.

### Proliferative Index (PI)

**Figure 3:**
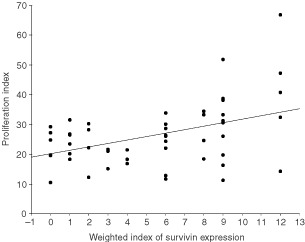
Increasing proliferative indices correlate significantly with rising weighted scores for Survivin expression in pancreatic cancers (*P*=0.001).

PI for pancreatic cancers was 26.2±10.5 %. There was a significant positive linear correlation between PI and Survivin weighted scores (*P*=0.001; Figure 3

). For ampullary cancers, PI was 33.3±13.2% and no correlation with Survivin expression was detected.

### TUNEL assay

**Figure 4:**
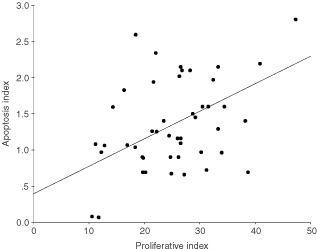
Significant positive correlation between proliferative and apoptosis indices in pancreatic cancers (*P*<0.001).

**Figure 5:**
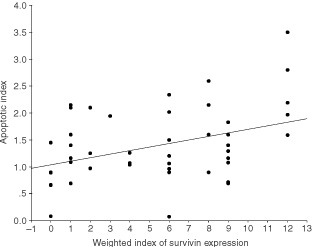
Increasing apoptosis indices correlate significantly with rising weighted scores for Survivin expression in pancreatic cancers (*P*=0.007).

The TUNEL assay labelled apoptotic bodies. Apoptosis index (AI) for pancreatic cancer was 1.38±0.69%. There were significant positive linear correlations between AI and PI (*P*<0.001; Figure 4

) and between AI and Survivin weighted score (*P*=0.007; Figure 5

) for pancreatic cancer. For ampullary cancer, AI was 1.59±0.62% and, in contrast to pancreatic cancers, there was a trend (not statistically significant) towards negative correlation with AI and Survivin weighted scores (data not shown).

### Survival characteristics

**Figure 6:**
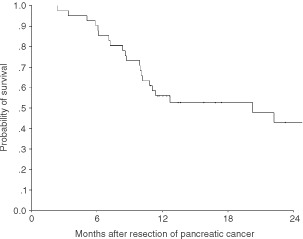
Kaplan–Meier analysis of survival following pancreaticoduodenectomy for pancreatic cancer.

Long-term follow up data were available for 41 patients with pancreatic cancer, none of whom had died in the post-operative (60 days) period. The disease-specific median survival period was 20.3 (95% confidence interval: 6.1–34.4) months with an actuarial survival rate of 55.8% at 1 year and 43.0% at 2 years (Figure 6

), with a median follow-up of 13.4 (3.8–82.2) months. Only one patient was alive at 5 years following operation. On univariate analyses, a positive resection margin was a significant predictor of death due to recurrent disease. Age >60 year, presence of nodal metastases or grade 3 differentiation also appeared to predict death but did not achieve statistical significance (Table 2

**Table 2:**
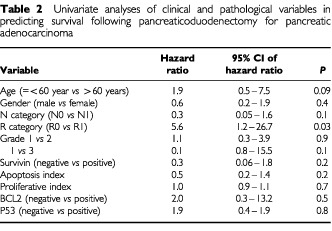
Univariate analyses of clinical and pathological variables in predicting survival following pancreaticoduodenectomy for pancreatic adenocarcinoma

). Survivin expression did not predict survival at any stratifying value of the weighted index. Similarly, a predictive value was not detected for indices of proliferation or apoptosis. Survival analyses were not conducted for ampullary and bile duct cancers because of the small numbers of patients.

## DISCUSSION

Expression of Survivin, of varying extent and intensity, was detected in 88% of adenocarcinomas of the pancreas. Non-neoplastic acinar parenchyma and pancreatic duct epithelium were negative. The prevalence of Survivin expression was similar to that reported in another smaller series of pancreatic cancers (26 cases; 77% positive) (Satoh *et al*, 2001). In contrast to previous observations (Adida *et al*, 1998b; Satoh *et al*, 2001), however, Survivin immunostaining was detected in a proportion of endocrine cells in islets of Langerhans. Using a similar polyclonal antibody, cytoplasmic Survivin expression has been described in 93% of malignant melanomas (Grossman *et al*, 1999a), 81% of basal cell carcinomas, 92% of cutaneous squamous cell carcinomas (Grossman *et al*, 1999b), 88% of gastric carcinomas (Okada *et al*, 2001) and 100% of oesophageal cancers (Beardsmore *et al*, 2001). Earlier studies with a monoclonal antibody report a generally lower prevalence of Survivin expression in 34% of gastric carcinomas (Lu *et al*, 1998), 53–61% of colorectal carcinomas (Kawasaki *et al*, 1998; Sarela *et al*, 2001), and 71% of breast carcinomas (Tanaka *et al*, 2000). Although Survivin was initially described as a ubiquitous and universally present marker of human epithelial malignancies (Ambrosini *et al*, 1997), such differences in the prevalence of expression may be explained by intrinsic differences in tumour biology and higher affinity of the polyclonal antibody as compared to its monoclonal counterpart (Altieri *et al*, 1999).

There was no correlation between Survivin expression and any clinical or pathological characteristic of pancreatic adenocarcinoma. A similar absence of correlation has also been noted in gastric (Lu *et al*, 1998), colorectal (Kawasaki *et al*, 1998; Sarela *et al*, 2001) and breast cancers (Tanaka *et al*, 2000), although Survivin expression is associated with a histologically more aggressive phenotype of neuroblastomas (Adida *et al*, 1998a) and transitional cell carcinomas of the bladder (Swana *et al*, 1999). In the present series, there was a high proportion of tumours at locally advanced stage and this reflects the authors' clinical practice and regional referral patterns. Such a distribution of pathological stages and the high prevalence of Survivin expression might have rendered the power of this study insufficient to demonstrate any correlation between Survivin expression and specific pathological characteristics.

Potential relationships between Survivin expression and cancer cell proliferation or apoptosis were explored. There was significant positive correlation between increasing weighted indices of Survivin expression and rising proliferative indices. A similar correlation between Survivin expression and proliferative index has also been reported for hepatocellular carcinomas (Ito *et al*, 2000). These observations are entirely consistent with the regulated expression of Survivin in the G2/M phase of the cell-cycle such that over-expression may overcome an apoptotic checkpoint and favour aberrant progression of malignant transformed cells through mitosis (Li *et al*, 1999). It is also known that anti-sense targeting of Survivin gene expression results in inhibition of cellular proliferation (Ambrosini *et al*, 1998), whereas Survivin over-expression promotes cell cycle entry with an accelerated S phase shift and resistance to G1 arrest (Suzuki *et al*, 2000).

Apoptosis indices were uniformly low and there was significant direct correlation between proliferative and apoptotic indices in pancreatic adenocarcinoma. Inhibition of apoptosis promotes aberrantly prolonged survival of mutated cells and such inhibition appears to be a central event in malignant transformation (Bedi *et al*, 1995). Furthermore, the correlation between proliferation and apoptosis is in keeping with a well-recognised biological paradigm, the ‘dual-signal model’, which proposes that the machineries mediating growth and apoptosis are coupled processes (Evan and Littlewood, 1998). In this model, activation of cell proliferation necessarily primes the cellular apoptotic programme that, unless countermanded by appropriate survival signals, automatically removes the cell.

A direct correlation between increasing indices of Survivin expression and rising apoptotic indices was also observed in pancreatic cancer. This was surprising given the established role of Survivin as an inhibitor of apoptosis (Ambrosini *et al*, 1997). In contrast to the present observation, an association between Survivin expression and reduced apoptotic indices has been reported in another series of pancreatic cancer (Satoh *et al*, 2001) and also for colorectal (Kawasaki *et al*, 1998), gastric (Lu *et al*, 1998), and breast cancer (Tanaka *et al*, 2000). In the present study, too, there was a trend towards an association between higher weighted scores of Survivin expression and lower apoptosis indices in the limited number of ampullary cancers. In hepatocellular carcinoma, however, no relation between Survivin expression and apoptosis has been detected (Ito *et al*, 2000). Such variability may be explained by tumour-specific intrinsic differences in the complex apoptotic regulatory cascade, which includes several other molecular regulatory factors important amongst which are P53 and BCL-2 (Guo and Hay, 1999).

In the present study, 54% of pancreatic cancers were P53-positive and this is consistent with previous reports of 47% (Bold *et al*, 1999), 54% (Sinicrope *et al*, 1996) and 64% (Hu *et al*, 1998). Although immunohistochemistry does not distinguish between wild-type and mutated or inactivated forms of P53, it is generally accepted that accumulated P53 represents a mutated type (Hall and Lane, 1994). Inactivation of P53 predisposes to dysregulated proliferation and aberrantly prolonged survival of mutated cells (Evan and Littlewood, 1998) and this is reflected in the high frequency of P53 inactivation in human cancers (Harris and Hollstein, 1993). There was a significant correlation between expression of Survivin and P53 in pancreatic cancer. Such an association has been reported in gastric cancer (Lu *et al*, 1998) and it is postulated that P53, in it's role as a transcriptional repressor (Levine, 1997) may negatively regulate Survivin gene expression by a mechanism that is disrupted by P53 mutation. Expression of BCL-2 was detected in 12% of pancreatic cancers and the sensitivity and specificity of immunostaining were confirmed by the prominent expression of BCL-2 in infiltrating lymphocytes in all cases examined. The expression of BCL-2 in pancreatic cancers is widely variable, with prevalence ranging from 0% (Evans *et al*, 2001) to 55% (Sinicrope *et al*, 1996).

Survival characteristics of the present cohort of patients with pancreatic cancers compare well with other series (Yeo *et al*, 1998). There was no association between survival and expression of Survivin. The relationship between Survivin expression and aggressiveness of disease is clearly complex. For example, in colorectal cancer Survivin expression predicts death due to recurrent disease specifically for patients with node-negative but not node-positive disease (Sarela *et al*, 2000, 2001) and not for colorectal cancer patients at all stages taken as a whole (Kawasaki *et al*, 1998). It has been previously argued that detection of Survivin expression may independently predict a worse outcome only in specific subgroups of patients with early stage-cancer (Sarela, 2000) and this may partly explain the absence of prognostic function for Survivin in this series of locally advanced pancreatic adenocarcinoma. Similarly, unlike colorectal (Kawasaki *et al*, 1998) or breast cancer (Tanaka *et al*, 2000) the apoptosis index, did not predict survival for patients with pancreatic adenocarcinoma.

Analysis of ampullary carcinomas revealed Survivin expression in a similar high percentage (83%) of cases. However, in contrast to pancreatic cancer, there was no correlation between Survivin expression and P53 or proliferative index, and there was a trend towards negative correlation between apoptosis and Survivin expression. Although based on analysis of only a small number of cases, these results further support the concept that adenocarcinom of the pancreatic head and ampullary region are separate entities with a different prognosis.

In summary, Survivin is expressed in the majority of pancreatic cancers and the extent of expression correlates strongly with proliferative and apoptotic functions of malignant cells. These results raise several exciting therapeutic and diagnostic possibilities. Firstly, inhibition of Survivin expression by anti-sense treatment has been reported to improve the effectiveness of chemotherapy (Olie *et al*, 2000) and might also impact on radiation therapy, especially in the light of experimental evidence that Survivin acts as a radio-resistance factor in pancreatic cancer cells (Asanuma *et al*, 2000). Secondly, diagnosis of pancreatic cancer by peripheral blood-examination for anti-Survivin antibodies, as in lung or colorectal cancer (Rohayem *et al*, 2000) or analysis of pancreatic duct cytology samples, analogous to urine detection of Survivin as a diagnostic test for bladder cancer, (Smith *et al*, 2001) may be possible. Finally, the extent of Survivin expression may help predict response to neoadjuvant or adjuvant chemotherapy, as reported for oesophageal cancer (Kato *et al*, 2001).
